# Emergency medicine education via the micro-course and flipped classroom-reform of medical education during the COVID-19 pandemic

**DOI:** 10.1097/MD.0000000000036459

**Published:** 2023-12-15

**Authors:** Cui Yang, Zheng-Wu Zhou, Long Jin, Lu Jiang, Sheng-Jin Han

**Affiliations:** a Department of Clinical Medicine, West Anhui Health Vocational College, Lu‘an, China; b Department of Emergency, Lu‘an Hospital of Anhui Medical University, Lu’an, China.

**Keywords:** emergency medicine, flipped classroom, micro-course, reform in education

## Abstract

The “micro-course” and “flipped classroom” are emerging tools for medical education but little is known about their utility for emergency medicine teaching. The suitability of the micro-course combined with flipped classroom is investigated for delivery of an emergency medicine course in West Anhui Health Vocational College. Students from Class A and Class B of the Clinical Department of West Anhui Health Vocational College, Grade 2000, were assigned to experimental (micro-course plus flipped classroom, n = 102) and control (traditional, didactic teaching, n = 104) groups, respectively. The effectiveness of teaching was assessed by theoretical tests and questionnaires at the end of the course. Theory test results were significantly better for the experimental group than for controls (*t* = 3.122, *P* < .01). General satisfaction of students who had participated in the micro-course plus flipped classroom exceeded that of those who had received traditional teaching. Enthusiasm, efficiency, and learning facility was self-reported to be enhanced by students in the experimental group relative to controls. Use of the micro-course combined with flipped classroom successfully increased the outcome of emergency medicine teaching and may be considered as an approach to reform emergency medicine teaching in medical colleges and universities.

## 1. Introduction

Clinical medical professionals who have received training in higher vocational colleges are required to execute the professional duties of judgment and treatment of patients with acute and critical diseases and to recognize the need to refer to an appropriate specialist. Emergency medicine is included in medical college undergraduate and professional teaching courses but some deficiencies in its teaching remain. Approaches that may be adopted to improve teaching include symptom-centered teaching, problem-solving, diversified methods, practical teaching, assessment reform and strengthening skills involved in operational training, humanistic education, clinical probation and practice.

Traditional classroom teaching in China generally involves one-way knowledge transfer with little student participation, fostering the passive acceptance of information by students and creating a dull classroom atmosphere during a prolonged teaching time. It is difficult to engage and retain the attention of students and such a mode of teaching does little to stimulate their enthusiasm and initiative. However, the utility of the flipped classroom (FC) has been recognized since 2000.^[1]^ The basic premise is that students arrive at the teaching venue having educated themselves on the topic under consideration, freeing up classroom time for discussion and question and answer activities.^[2]^ Independent preparation on the part of the student is required, discussion is promoted and teacher-student interactions encouraged.^[3]^

However, use of the FC requires students to invest considerable time in preparation,^[4–7]^ classroom teaching content is not always well-understood and actual clinical cases may differ from the ones they have studied.^[1,4,6,8]^ It has been suggested that combining the micro-course with a FC approach may overcome some of these problems.^[9]^

The micro-course describes a short video based on online resources watched by students before class that summarizes relevant points of knowledge.^[10]^ The simplicity of the approach appeals to college students and a micro-course and FC combination has been widely applied to medicine and other majors.^[11–13]^ The micro-course/FC combination was applied to the interdisciplinary emergency medicine course during the current study. The success of this approach was evaluated via final examination and a questionnaire and compared with traditional teaching methods.

## 2. Subjects and methods

### 2.1. Sample

College students of Grade 2020 at the Department of Clinical Medicine of West Anhui Health Vocational College were enrolled. Class A (n = 102) were assigned to an experimental group (micro-course/ FC) and Class B (n = 104) to a control group (traditional teaching). Students are routinely assigned to classes in order of enrollment at the college, all courses followed the syllabus and emergency medicine was taught by the same teacher throughout. Ethical approval was granted by the Ethics Committee of West Anhui Health Vocational College and all participants gave written informed consent.

### 2.2. Design and application of micro-course and FC

A micro-video was created based on each chapter of Emergency Medicine (4th edition) published by the People’s Health Publishing House, PR China. A total of 17 points of knowledge points were incorporated into micro-videos to satisfy syllabus requirements, totaling 120 minutes (Table 1).

**Table 1:** Contents of teaching and micro-videos.

Chapter	Title	Micro-video	Video length
2	Cardiopulmonary-cerebral resuscitation	CPR operation and Use of defibrillator	10 min
3	Shock	Deep venipuncture for catheterization	10 min
4	Fever	First aid treatment for febrile convulsions	10 min
5	Expiratory dyspnea	Use of ventilator	5 min
6	Disorder of consciousness	First aid procedures for stroke patients	5 min
7	Acute pain	First aid procedures for acute chest pain	10 min
7	Acute pain	First aid process for acute abdominal pain	10 min
7	Acute pain	Abdominal paracentesis	5 min
9	Hyperspasmia	First aid treatment for epilepsy	5 min
11	Oligouria, anuria and urinary retention	The management of urinary retention	5 min
12	Trauma first aid	Intratracheal intubation technique	5 min
12	Trauma first aid	Chest closed cavity drainage	10 min
12	Trauma first aid	Debridement and suturing	5 min
12	Trauma first aid	Application of bedside ultrasound	5 min
13	Acute poisoning	Gastrolavage	5 min
14	Injury of environmental and physicochemical factors	First aid treatment of a venomous snake bite	5 min
15	Dibaster assistance	First aid treatment of the batch wounded	10 min

CPR = cardiopulmonary resuscitation

### 2.3. Pre-class

Micro-course videos were uploaded to the Chao-xing Campus Online Teaching Platform and students told to watch and learn prior to class. Students were able to leave questions on the platform for teachers to address during the classroom time.

### 2.4. Classroom time

Students signed in and points from the previous session were reviewed. The teacher addressed the questions left by students on the online platform. The teacher introduced clinical cases and issues based on the content of the micro-videos for classroom discussion by groups of students among themselves. A session during which the teacher led the students to consolidate their conclusions and which was oriented towards problem-solving followed. This teaching episode embodied the principles of the FC. The session was rounded off with a brief test which students filled in on their own to assess their facility with the material presented.

### 2.5. Traditional education methods

The control group were required to prepare material from the syllabus in advance and received didactic teaching with homework assigned for completion after class.

### 2.6. Impact assessment

Emergency Medicine is a compulsory course, assessed by closed-book examination for a final percentage mark. Examination papers were set by the teaching and research group and marked by the teachers. Scores were collected for statistical analysis.

A questionnaire, designed in accordance with preliminary research guidelines, was distributed to students after the end of the course.^[14,15]^

### 2.7. Statistical methods

SPSS 19.0 software (SPSS, Inc., Chicago, IL) was used for statistical analyses. Count data are expressed as number of cases (n, %), using the 4-grid table *χ*^2^ test. Quantitative data are expressed as mean ± standard deviation. Comparisons between the 2 groups were made using an independent sample *t* test. A value of *P* < .05 was considered to be statistically significant.

## 3. Results

### 3.1. Theory test

**Figure 1.**
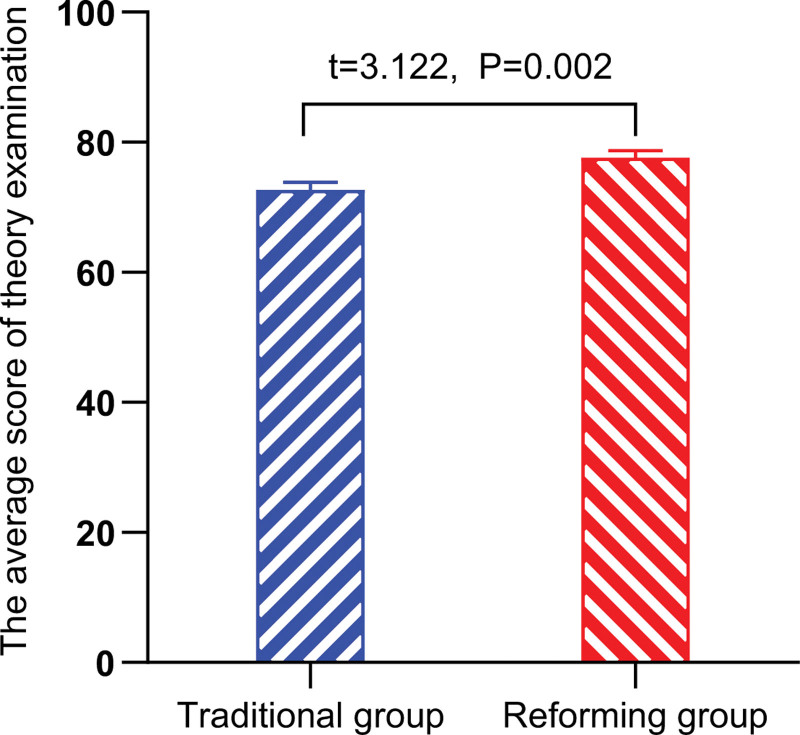
The average score of theory examination between the 2 groups.

The students’ facility with basic theories was assessed by means of a test. The mean score for the experimental group was significantly higher at 77.64 ± 7.56 than that of the control group, 72.70 ± 8.45 (*t* = 3.122, *P* = .002, Fig. 1).

### 3.2. Students’ questionnaire

A total of 206 questionnaires were distributed and 98.06% (202/206) were recovered with 1 questionnaire lost from each group. 78.43% of students in the experimental group felt that the micro-course/FC stimulated their learning interest in emergency medicine (Table 2); 74.51% felt it promoted their understanding and theoretical knowledge and 74.51% found it helpful with communication skills. The corresponding percentages for the control group were 57.69%, 50.00%, and 51.92%.

**Table 2:** Outcomes of students’ questionnaire.

Statement number	Statement	Experimental group (n = 102)			Control group (n = 104)			*χ* ^2^	*P* value
		VS/S (%)	U (%)	D/VD (%)	VS/S (%)	U (%)	D/VD (%)		
Q1	Stimulation of interest in learning	78.43 (80/102)	11.76 (12/102)	9.81 (10/102)	57.69 (60/104)	23.08 (24/104)	19.23 (20/104)	6.316	0.043
Q2	Promotion of understanding of theoretical knowledge	74.51 (76/102)	11.76 (12/102)	13.73 (14/102)	50.00 (52/104)	22.12 (23/104)	27.88 (29/104)	6.911	0.032
Q3	Help with communication skills	74.51 (76/102)	11.76 (12/102)	13.73 (14/102)	51.92 (54/104)	30.77 (32/104)	17.31 (18/104)	7.41	0.025
Q4	Enhancement of capacity for self-study	70.59 (72/102)	15.68 (16/102)	13.73 (14/102)	57.69 (60/104)	22.12 (23/104)	20.19 (21/104)	2.009	0.366
Q5	Improvement of analysis and problem solving	76.47 (78/102)	9.80 (10/102)	13.73 (14/102)	63.46 (66/104)	9.62 (10/104)	25.00 (26/104)	3.069	0.216

D = dissatisfied, Q1~5 = question 1~5, S = satisfied, U = uncertain, VD = very dissatisfied, VS = very satisfied.

Students receiving teaching via the micro-course/FC showed significantly higher levels of satisfaction (*P* < .05). The above results revealed that the new model was also more efficient in developing students’ aggressiveness and competence. However, it should be noted that there was no statistical difference between the experimental and control groups for responses to Q4 and Q5.

## 4. Discussion

Emergency medicine is a secondary discipline, requiring knowledge of all the systems of the body. Emergency patients change rapidly, often against a background of a critical condition. Emergency doctors must assess vital signs and consciousness quickly and give timely and appropriate treatment for life-threatening conditions. Medical students generally learn the complicated emergency medicine teaching content poorly and health vocational college is often approached from a low starting point with low capacity for independent study and understanding. Students commonly learn by rote, failing to develop powers of summary and analysis, and lack enthusiasm and initiative.

Most higher vocational colleges rely on didactic teaching with heavy clinical burdens precluding much interaction between staff and students. The multiple systems referred to during emergency medicine teaching make the subject onerous and boring.^[16]^ The need for teaching innovation and reform is appreciated by many medical colleges and universities. The micro-course/FC investigated during the current work allows students to express their thoughts, increasing the efficiency of study. A micro-video linked to internet resources was created for the study and used in combination with the FC.

The FC education model has been shown to benefit the teaching of pharmacology,^[10]^ neurosciences,^[17]^ radiology,^[2]^ physical education health,^[18]^ and entrepreneurship.^[19]^ Improvements in students’ study skills are shown and communication between teachers and students enhanced. The pre-class micro-course overcame the shortness of the teaching time, freeing classroom sessions for higher-level discussions. Such student-centered education elicited more positive feedback from students. Post-course examination gave higher marks for the experimental than control group of the present study (*P* < .05) showing promise for the mastery of clinical skills among the students involved.

The current study shows that the novel educational mode of micro-course/FC has utility for emergency medicine education. Basic concepts must be mastered and clinical emergency skills acquired and this novel teaching approach can help students realize this target. Students acquire the basics of first-aid procedures through the micro-video, freeing classroom time for analysis of typical clinical cases. Teachers may devote more time to applying clinical first aid skills to clinical practice to aid clinical first aid thinking. Many students said that their grasp of emergency treatment and clinical diagnosis had improved and their confidence in engaging with treatment discussion during clinical clerkship teaching had been enhanced.

## 5. Conclusion

In summary, the micro-course/FC is a useful supplement to traditional methods in emergency medicine education. From the results of this article, we can see that through the education reform, students’ interest in learning and subjective initiative have been significantly improved, and the new teaching methods can be recognized by the majority of students, which is also a change made in the face of the impact of the global epidemic. However, this research also exist some limitations. In this study, only 2 classes are included, which is small in number and short in time. There may be statistical bias. In addition, this reform has not been recognized by the majority of experts, and whether it can be tested for a long time remains to be tested. Extension of the approach to entire grades and departments is required to explore its effectiveness further.

## Author contributions

**Formal analysis:** Long Jin.

**Funding acquisition:** Cui Yang.

**Investigation:** Cui Yang.

**Resources:** Lu Jiang.

**Software:** Long Jin.

**Supervision:** Zheng-Wu Zhou.

**Writing – original draft:** Sheng-Jin Han.

**Writing – review & editing:** Sheng-Jin Han.
